# HDAC Inhibitors Disrupt Programmed Resistance to Apoptosis During *Drosophila* Development

**DOI:** 10.1534/g3.117.041541

**Published:** 2017-04-27

**Authors:** Yunsik Kang, Khailee Marischuk, Gina D. Castelvecchi, Arash Bashirullah

**Affiliations:** * Division of Pharmaceutical Sciences, University of Wisconsin-Madison, Wisconsin 53705-2222; † Laboratory of Genetics Graduate Program, University of Wisconsin-Madison, Wisconsin 53705-2222

**Keywords:** apoptotic thresholds, caspase activation, TSA, SAHA, HDAC1/Rpd3

## Abstract

We have previously shown that the ability to respond to apoptotic triggers is regulated during *Drosophila* development, effectively dividing the fly life cycle into stages that are either sensitive or resistant to apoptosis. Here, we show that the developmentally programmed resistance to apoptosis involves transcriptional repression of critical proapoptotic genes by histone deacetylases (HDACs). Administration of HDAC inhibitors (HDACi), like trichostatin A or suberoylanilide hydroxamic acid, increases expression of proapoptotic genes and is sufficient to sensitize otherwise resistant stages. Conversely, reducing levels of proapoptotic genes confers resistance to otherwise sensitive stages. Given that resistance to apoptosis is a hallmark of cancer cells, and that HDACi have been recently added to the repertoire of FDA-approved agents for cancer therapy, our results provide new insights for how HDACi help kill malignant cells and also raise concerns for their potential unintended effects on healthy cells.

Resistance to apoptosis has been extensively characterized in the context of cancer; an acquired trait that makes malignant cells impervious to endogenous and therapeutic attempts to initiate apoptosis (Hanahan and Weinberg 2000, 2011). Mechanistically, cancer cells evade apoptosis either through disruptions in the ability to detect cellular damage that should initiate apoptosis, as occurs with loss of the DNA damage sensor *p53*, or through disruptions in the ability to execute apoptosis (Hanahan and Weinberg 2000, 2011). Defects in execution often occur as a result of decreased levels of proapoptotic and/or increased levels of antiapoptotic factors (Lowe *et al.* 2004; Adams and Cory 2007). These alterations in the overall balance of apoptotic regulators increase the threshold required to trigger apoptosis, effectively making malignant cells more resistant to apoptotic stimuli.

Conventional chemotherapy relies heavily on the use of cytotoxic agents that target the DNA and cytoskeleton of all cells in order to trigger apoptosis in tumor cells. Development of new classes of therapeutic agents has been driven by the growing evidence for the role of epigenetics in cancer (West and Johnstone 2014). As a result, one of the most promising pharmacological agents is small molecule inhibitors of histone deacetylases (HDACs). HDACs are critical regulators of chromatin remodeling, removing acetylation marks on histone proteins within nucleosomes and thereby promoting “closed” chromatin conformations that lower rates of transcription (Haberland *et al.* 2009). By opposing this activity, HDAC inhibitors (HDACi) effectively increase the rate of transcription. Most HDACi are reversible inhibitors with a wide range of potencies, from nanomolar range for trichostatin A (TSA) to micromolar range for others like suberoylanilide hydroxamic acid (SAHA) (de Ruijter *et al.* 2003). SAHA was the first HDACi to be approved for cancer therapy, but more are currently in clinical trials (Marks and Breslow 2007; West and Johnstone 2014). TSA also appears to have potentially beneficial therapeutic outcomes for breast cancer (Vigushin *et al.* 2001), but its high potency also makes it more toxic. Although most HDACi target broad classes of HDACs, their therapeutic potential relies heavily on their capacity to promote apoptosis (Bolden *et al.* 2006; West and Johnstone 2014; Newbold *et al.* 2016). This proapoptotic effect is generated by increasing expression of proapoptotic genes, which, in turn, lowers the apoptotic threshold (Bolden *et al.* 2006; Newbold *et al.* 2016). Historically, this HDACi-mediated sensitization toward apoptosis was thought to be selective to tumor cells (Dokmanovic and Marks 2005; Bolden *et al.* 2006); however, the mechanisms for this selectivity, if true, are unclear.

Many healthy cells also exhibit properties that resemble the resistance to apoptosis observed in cancer cells. Adult mammalian cells in the kidney and brain, for example, are highly resistant to apoptotic stimuli, while those in the bone marrow and thymus are highly sensitive to the same treatment (Ni Chonghaile *et al.* 2011). These differences in the ability to respond to apoptotic stimuli are also dynamically regulated during development. Developing neurons, for example, are very sensitive to apoptotic stimuli, while mature neurons are highly resistant to the same apoptotic insults (Kole *et al.* 2013). The acquisition of this resistance during neuronal differentiation is accompanied by transcriptional repression of a proapoptotic gene, the caspase adaptor protein Apaf-1 (Wright *et al.* 2004, 2007). The similarities between this developmental program and its counterpart phenomena in cancer raise the possibility that the mechanisms of programmed resistance are co-opted during tumorigenesis.

We have previously shown that the ability to respond to apoptotic triggers is regulated during the *Drosophila* life cycle (Kang and Bashirullah 2014). Some stages during development are 50 times more resistant to lethal apoptotic stimuli, like ultraviolet radiation or expression of the IAP-antagonist *reaper*, than other stages. Thus, while a strong apoptotic stimulus triggers an overwhelming caspase cascade and subsequent lethality at a sensitive stage, the same stimulus has no effect at a resistant stage. These permissive and restrictive developmental windows provide an ideal experimental context for studying mechanisms that establish and regulate resistance to apoptosis. Here, we examine if these observed differences in resistance to apoptosis are mediated by changes in levels of proapoptotic and/or antiapoptotic genes; moreover, given that HDACi are thought to selectively increase proapoptotic gene expression in cancer, we also test if HDACi feeding can alter the apoptotic threshold of tissues protected by programmed resistance during normal development.

## Materials and Methods

### Fly stocks

The Bloomington *Drosophila* Stock Center provided the following stocks: *Sgs3-GFP*, *dronc^51^*, *HDAC1^04556^*, *engrailed-Gal4*, *UAS-RFP*, *nubbin-Gal4*, *tubulin-Gal4*, *UAS-GFP*, *Df(3L)BSC673*, *Df(2R)BSC359*, *Df(3R)BSC547*, *Df(2R)BSC785*, *Df(2R)ED1484*, *Df(3R)ED10642*, *Df(2R)BSC153*, *UAS-HDAC1 RNAi*, *UAS-HDAC3 RNAi*, and *w^1118^*. The following stocks were kindly provided by the fly community: *drice^Δ1^* (Muro *et al.* 2006), *dark^82^* (Akdemir *et al.* 2006), *hs-reaper* (White *et al.* 1996), and *HDAC3^N^* (Zhu *et al.* 2008). The control stock used was *w^1118^* and the other stocks were crossed into this background.

### Developmental staging

Early and wandering third instar larvae (eL3 and wL3, respectively) were sorted as previously reported (Kang and Bashirullah 2014). The mid-L3–specific reporter used was a GFP-tagged glue protein transgene (Sgs-GFP) (Biyasheva *et al.* 2001). We used this reporter to synchronize third instar (L3) development at the mid-L3 transition and generate a more detailed time course of L3 larvae. For eL3 animals, embryos were aged at 25° until 72–76 hr after egg laying (AEL) to collect animals that have recently molted into L3; 16 hr later, 88–92 AEL larvae that were not expressing Sgs3-GFP were used for the second time point. After this point, animals were monitored for Sgs3-GFP expression and collected in 4-hr increments. Other stages during the fly life cycle were aged at 25° from egg laying for embryonic (0–6, 6–12, and 12–18 hr AEL) and larval stages (L1: 30–42 hr AEL, L2: 54–66 hr AEL), or from puparium formation for stages during metamorphosis.

### Delivery of apoptotic activators and survival assays

Assessment of survival after delivery of apoptotic triggers was performed using previously described methods (Kang and Bashirullah 2014). Transgenic lines with the death activator *reaper* directly fused to the hsp70 heat-shock promoter [*hs-reaper* (White *et al.* 1996)] were used in all experiments. Appropriately staged animals were heat-shocked by submerging grape agar plates sealed with Parafilm in a water bath at 37° for 30 min; after heat-shock, animals were transferred to agar plates at 25°. This heat-shock treatment results in ∼120-fold expression of *reaper* in animals carrying one copy of the *hs-reaper* transgene and ∼350-fold expression in animals carrying two copies of the *hs-reaper* transgene (Kang and Bashirullah 2014). The survival curves were assessed by hatching in embryos, touch response in larvae, heartbeat in prepupae, head eversion in pupae, and eclosion for completion of development. Every experiment was done with three or more replicates of 25–35 animals each, and the error bars in the figures represent SD.

### Quantitative real-time PCR

Transcript levels of target genes were measured by quantitative real-time PCR (qPCR) using previously described methods (Ihry *et al.* 2012). RNA was isolated from appropriately staged animals using the RNeasy Plus Mini Kit (Qiagen). cDNA was synthesized from 200 to 400 ng of total RNA using the SuperScript III First-Strand Synthesis System (Invitrogen). qPCR was performed on a Roche 480 LightCycler using the LightCycler 480 DNA SYBR Green I Master kit (Roche). In all cases, samples were run simultaneously with three independent biological replicates for each target gene, and *rp49* was used as the reference gene. To calculate changes in relative expression, the Relative Expression Software Tool was used (Pfaffl *et al.* 2002). For absolute quantification, the Ct (threshold cycle) for each target gene was compared to a standard curve generated for Ct’s of the respective amplicons at known concentrations.

### Immunofluorescence and image capture

Wing imaginal discs from appropriately staged wL3 animals were fixed and immunostained using previously reported methods (Yin *et al.* 2007). The primary antibodies used were rabbit α-cleaved caspase-3 (1:200; Cell Signaling), rabbit α-cleaved Dcp-1 (1:200; Cell Signaling), and rabbit α-Drice (1:500; a gift from P. Friesen). Secondary antibodies used were Alexa Fluor 488 α-rabbit (1:200; Invitrogen) and Cy3 α-rabbit (1:200; Jackson ImmunoResearch Laboratories). Images were taken on an Olympus FluoView FV1000 confocal microscope and optimized with the FV10-ASW software.

### HDACi feeding

HDACi was added to the fly food as previously described (Pile *et al.* 2001). A total of 10 ml boiled hot cornmeal molasses food was added to each individual vial. The vials were submerged in a 55° water bath to cool down. Once cooled, the appropriate amount of TSA (Sigma) or SAHA (LC Laboratories) was added and vortexed vigorously. Roughly 150 embryos were placed on the food and aged at 25° until the animals were collected at the desired developmental stage.

### Data availability

All *Drosophila* stocks used in this study are available upon request. All data necessary for confirming the conclusions presented are represented fully within the article.

## Results

### Developmentally programmed resistance to apoptosis is tightly correlated with reduced levels of proapoptotic genes

**Figure 1:**
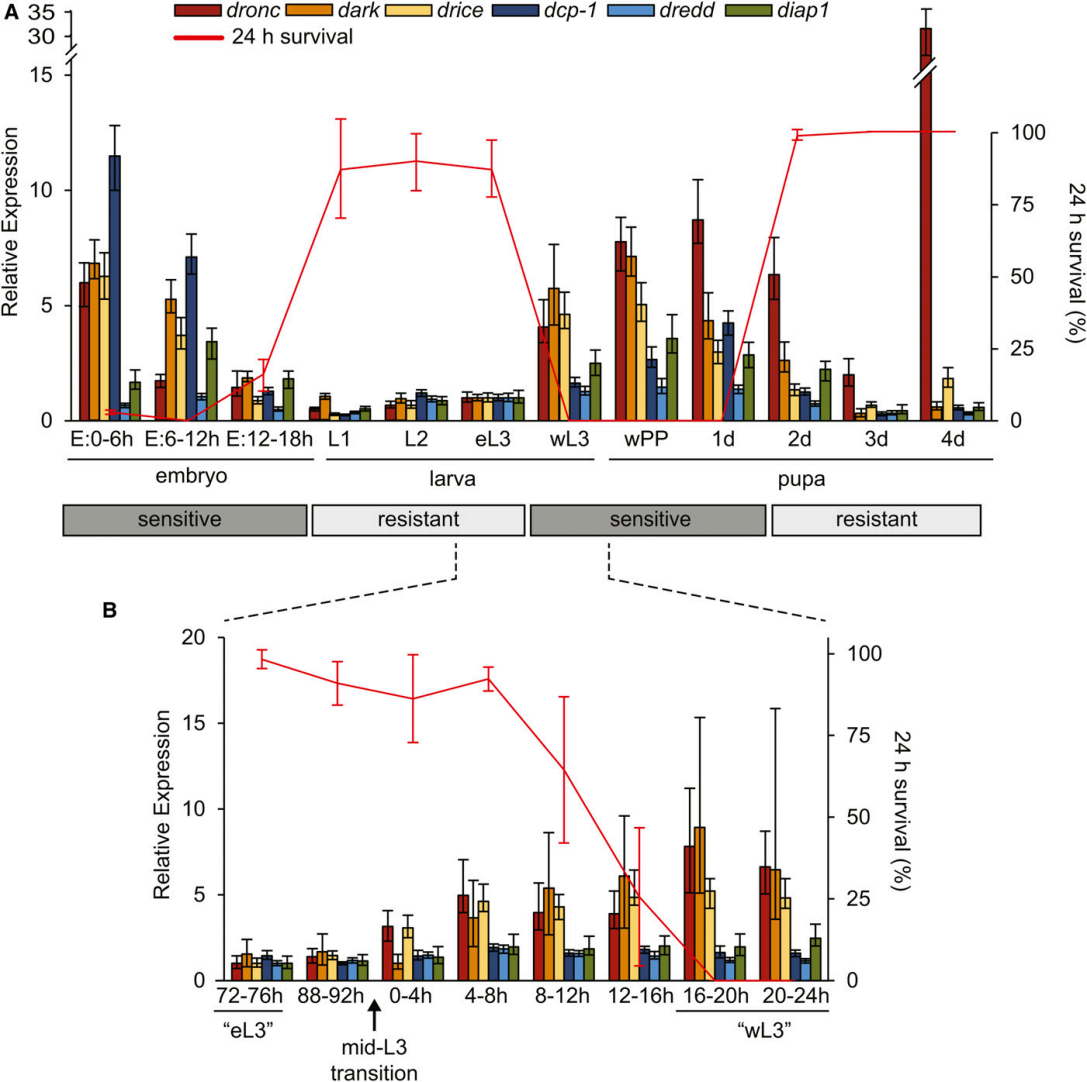
Programmed resistance to apoptosis during *Drosophila* development is tightly correlated with levels of proapoptotic genes. (A) Levels of proapoptotic genes and 24-hr survival after *reaper* treatment across the fly life cycle. Twelve stages were examined: three embryonic stages (in 6-hr windows after egg laying), four larval stages (first, second, and third instars; the latter stage is twice as long and is divided into early and wandering third-instar stages), and four pupal stages (white prepupae “wPP” defining the onset of metamorphosis, followed by four sequential 1-d stages). The red line represents 24-hr survival rates for each stage after a ∼120-fold induction of the IAP-antagonist *reaper* (*y*-axis on the right side). Bar graphs represent mRNA levels of critical apoptotic genes (*y*-axis on the left side), measured by qPCR for four caspases (*dronc*, *drice*, *dcp-1*, and *dredd*), the Apaf-1 caspase adaptor *dark*, and the antiapoptotic inhibitor of apoptosis protein *diap1*. (B) Levels of proapoptotic genes and 24-hr survival after *reaper* treatment across third-instar development. As in (A), the red line represents 24-hr survival (*y*-axis on the right side) and the bar graphs represent levels of apoptotic genes measured by qPCR (*y*-axis on the left side). Before the mid-L3 transition, animals are staged by hours after egg laying; after the mid-L3 transition, animals are staged by hours after Sgs-GFP expression (see *Materials and Methods*). For the 24-hr survival assay, each condition was tested in triplicate with at least 25 animals. The qPCR results reflect triplicate biological samples, represented relative to the levels of *rp49*, and then normalized to the corresponding levels in eL3 stage for (A) and to the stage with the lowest levels in (B). Error bars in qPCR calculated by Relative Expression Software Tool analysis and in survival curves reflect SD.

We have previously shown that the ability to trigger apoptosis is regulated during the *Drosophila* life cycle, with animals in some stages appearing impervious to apoptotic stimuli that are sufficient to kill animals at other stages (Kang and Bashirullah 2014). To determine if these differences in sensitivity to apoptosis correlated with corresponding changes in the basal expression of proapoptotic genes, we measured the sensitivity to apoptosis and mRNA levels of critical proapoptotic genes during development. We examined three embryonic, four larval, and five pupal stages, dividing the fly life cycle into 12 representative stages. To measure sensitivity to apoptosis, we tested the ability to survive a ∼120-fold induction of the IAP-antagonist *reaper*, generated by a 30-min heat-shock treatment in animals carrying one copy of the *hs-reaper* transgene (see *Materials and Methods* for details). The resulting 24-hr survival rates illustrate a contrasting landscape of sensitivity to apoptosis during development, with some stages showing lethality and others showing resistance (red line, Figure 1A). Animals during embryonic stages or those within 1 d of puparium formation died in response to *reaper*. In contrast, animals during the first 3 d of larval development or the last 3 d of pupal development were resistant, with >90% surviving the expression of *reaper*. These results are consistent with our previous observations that measured eclosion rates after expression of *reaper* (Kang and Bashirullah 2014), indicating that the lethal effects of *reaper* expression occur within the first 24 hr. Importantly, these results show that, during most of development, *Drosophila* are highly resistant to apoptotic triggers.

To measure levels of proapoptotic genes, we extracted total mRNA from each of the 12 representative stages defined above and performed qPCR for four caspases (*dronc*, *drice*, *dcp-1*, and *dredd*), the Apaf-1 caspase adapter *dark*, and the *Drosophila* inhibitor of apoptosis protein *diap1* (Figure 1A). The levels of *dredd*, a caspase primarily attributed to immune-related functions (Leulier 2000), do not vary significantly during development. However, the levels of *dronc*, *dark*, *drice*, and *dcp-1* are nearly fivefold lower in resistant stages during development. The one exception to this generalization occurs in late embryos (E: 12–18 hr) when levels of these proapoptotic genes are low even though these embryos are sensitive to apoptosis. This discrepancy is likely due to perdurance of protein products derived from the maternally deposited transcripts detected in the early embryo (see E: 0–6 hr in Figure 1A). In 2- and 4-d-old pupae (2 and 4 d, Figure 1A), the increased levels of *dronc* likely reflect the high tissue-specific expression in developing fat body and ovaries, respectively (Dorstyn *et al.* 1999; modENCODE Consortium *et al.* 2010). It is unclear if those tissues are destroyed by *reaper* treatment at those stages. Taken together, these results indicate a strong correlation between low levels of proapoptotic genes and resistance to apoptosis. Moreover, given that we have previously shown that loss of the antiapoptotic gene *diap1* does not trigger death in eL3 animals (Kang and Bashirullah 2014), it is not surprising that levels of *diap1* do not correlate with programmed resistance. In fact, levels of *diap1* appear to be higher during sensitive stages and lower during resistant stages (Figure 1A), suggesting that *diap1* likely serves to counteract the increased levels of proapoptotic caspases in sensitive stages rather than conferring resistance to apoptosis in resistant stages.

To further understand the relationship between resistance and levels of proapoptotic genes, we examined these two parameters during the global switch in sensitivity that occurs during L3 development. eL3 animals are resistant while wL3 are very sensitive (Figure 1, A and B). This global switch in sensitivity depends on the mid-L3 transition (Kang and Bashirullah 2014), a major developmental transition that is required for competence to enter metamorphosis and is associated with significant changes in gene expression (Riddiford 1993). One conspicuous change in gene expression during the mid-L3 transition is the appearance of mucin-like “glue” proteins in larval salivary glands (Beckendorf and Kafatos 1976; Korge 1977), which can be easily visualized in live animals carrying a Sgs3-GFP fusion transgene (Biyasheva *et al.* 2001). We used the appearance of Sgs3-GFP to synchronize animals at the start of the mid-L3 transition and divide the ensuing development in 4-hr increments. Calculation of absolute number of mRNA transcripts in eL3 shows that, at the onset of eL3 development (72–76 hr AEL), when animals are highly resistant to apoptosis, the levels of apoptotic genes are low but not off (Figure 1B and Supplemental Material, Figure S1 in File S1). After the mid-L3 transition, the levels of the proapoptotic genes *dronc*, *dark*, and *drice* gradually increase in expression until reaching their highest levels ∼16–20 hr later in wL3 animals. Strikingly, during this same period, there is a corresponding gradual loss in resistance to apoptosis. This gradual decrease in the 24-hr survival is also reflected in the gradual decrease in eclosion rates (Table S1 in File S1). After the start of the mid-L3 transition, animals that survive 24 hr also survive to eclose as adults; however, as the sensitivity to apoptosis gradually increases, a greater fraction of animals fail to eclose, likely a result of being unable to repair the increasing damage sustained during the expression of *reaper* (Table S1 in File S1). Taken together, these results demonstrate that sensitivity to apoptosis and levels of critical proapoptotic genes appear to be exquisitely proportional to each other, suggesting a causal relationship between transcriptional control of proapoptotic genes and programmed resistance to apoptosis.

### Reducing levels of critical proapoptotic genes is sufficient to confer resistance to apoptosis

**Figure 2:**
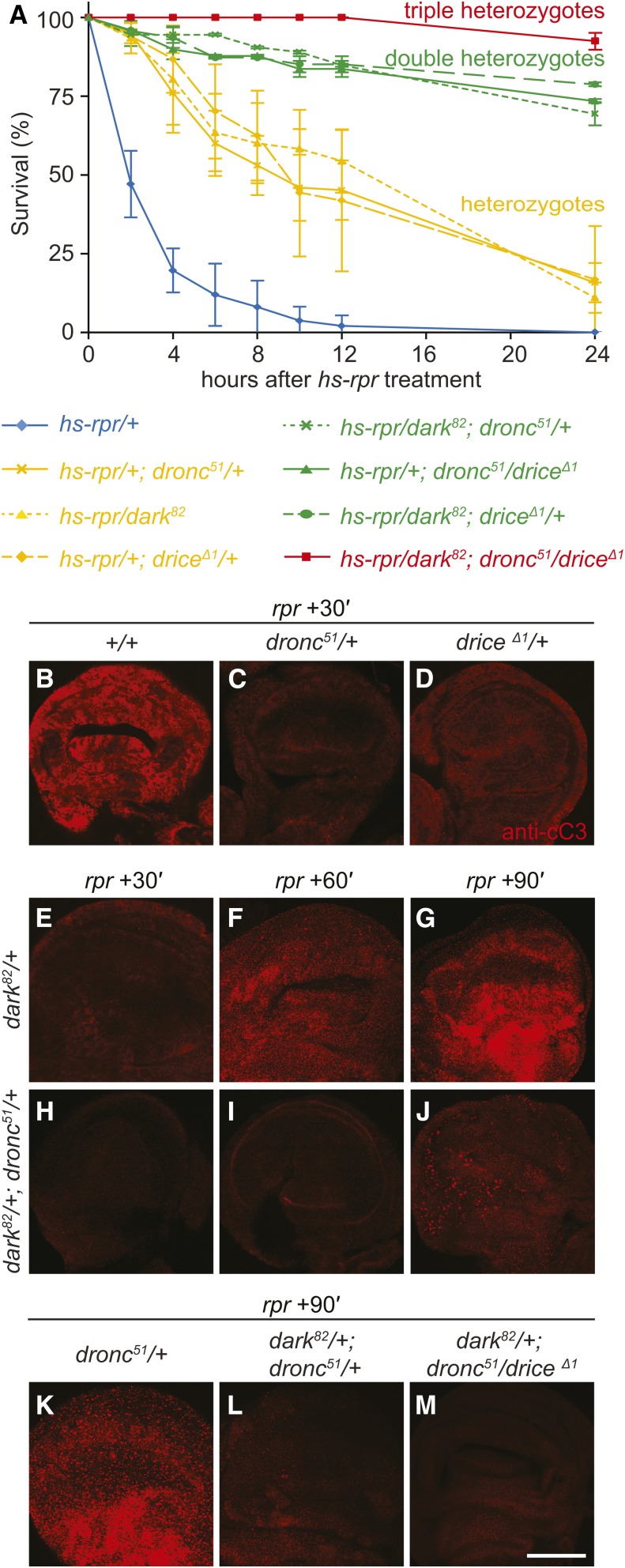
Reducing levels of proapoptotic genes is sufficient to confer resistance to apoptosis. (A) Survival curves for *reaper*-treated wL3 animals carrying null mutations in the proapoptotic genes *dronc*, *drice*, and/or *dark*. Genotypes of animals tested are as shown below and color coded as follows: control animals in blue, those carrying one heterozygous mutation in yellow (heterozygotes), those carrying two heterozygous mutations in green (double heterozygotes), and those carrying three in red (triple heterozygotes). Each condition was tested in triplicate with at least 25 animals each. Error bars reflect SD. (B–M) Caspase activation assays in wing imaginal discs dissected at 30, 60, or 90 min after *reaper* treatment in wL3 animals. Relative intensity of staining with antibodies directed to cleaved caspase-3 (anti-cC3) shows extent of caspase activation in each of the single, double, or triple heterozygous animals [genotypes same as those in (A)]. (B–D) Caspase activation 30 min after *reaper* treatment in wing discs dissected from control and *dronc* or *drice* heterozygous animals. (E–J) 30, 60, and 90 min time course in wing discs heterozygous for *dark* (E–G) or double heterozygous for *dark* and *dronc* (H–J). (K–M) Caspase activation 90 min after *reaper* treatment in wing discs dissected from single, double, or triple heterozygous animals. All images captured with the same imaging parameters. Bar, 100 µm.

To examine the causal relationship between levels of proapoptotic genes and programmed resistance to apoptosis, we tested the effect of losing one copy of *dronc*, *dark*, and/or *drice* in single, double, or triple heterozygous animals. We chose these three genes because they change the most between sensitive and resistant stages (Figure 1). By tracking survival over 24 hr, we show that animals heterozygous for these genes respond differently to the same pulse of *reaper*. In control wL3 animals, *reaper* expression kills animals very quickly, with 80% dead within 4 hr and nearly 100% dead within 12 hr (Figure 2A, blue line). Heterozygous animals carrying a null mutation in *dronc*, *dark*, or *drice* die more slowly, even though they still die within 24 hr (Figure 2A, yellow lines). In contrast, most double heterozygous animals survive 24 hr after the same treatment (Figure 2A, green lines), with about a third eclosing as adults (Table S2 in File S1). Each of the three single heterozygous genotypes and each of the three double heterozygous genotypes showed virtually identical survival curves (Figure 2A), suggesting that levels of these genes play an equivalent role in determining the global sensitivity to apoptosis. Strikingly, triple heterozygous animals were extremely resistant to *reaper*, with ∼90% surviving 24 hr and two-thirds eclosing as adults (Figure 2A, red line; Table S2 in File S1). These results demonstrate that even a twofold reduction in the levels of critical proapoptotic genes is sufficient to dramatically alter the ability to trigger apoptosis.

Next, to understand the effect of reducing levels of proapoptotic genes on the probability of triggering apoptosis, we measured the time course of caspase activation in the single, double, and triple heterozygous animals described above. Wing imaginal discs were dissected from *reaper*-treated wL3 animals and stained with antibodies directed to cleaved caspase-3 (anti-cC3). As expected, control wing discs at this sensitive stage show robust anti-cC3 staining within 30 min of *reaper* treatment (Figure 2B). However, this caspase activation is delayed in tissues heterozygous for either *dronc*, *dark*, or *drice*, showing significantly reduced activation of caspases 30 min after *reaper* treatment (Figure 2, C–E). Caspase activation does eventually occur in these heterozygous animals, but is delayed by 30–60 min (Figure 2, F, G, and K). Importantly, double and triple heterozygous wing discs show significantly reduced anti-cC3 staining even 90 min after *reaper* treatment (Figure 2, H–J, L, and M). These results indicate that twofold changes in the levels of critical proapoptotic genes can disrupt the onset of caspase activation and, as a result, the survival outcome in response to an otherwise to lethal dose of *reaper*.

### HDACi relieve programmed resistance to apoptosis

**Figure 3:**
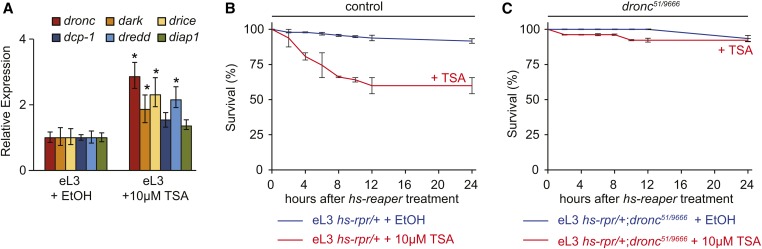
TSA relieves programmed resistance to apoptosis in eL3 animals. (A) Levels of apoptotic genes in eL3 animals that have been fed either the solvent carrier ethanol (EtOH) or 10 µM TSA. The apoptotic genes examined are the same as those in Figure 1. The qPCR results reflect triplicate biological samples, represented relative to the levels of *rp49*, and normalized to the control animals. Error bars were calculated by Relative Expression Software Tool analysis. *p < 0.05. (B and C) Survival curves for control (B) or *dronc* mutant (C) eL3 animals that have been fed either ethanol (blue lines) or 10 µM TSA (red lines), subjected to *reaper* expression, and then followed for 24 hr. Genotypes are shown below each of the graphs and each condition was tested in triplicates with at least 25 animals each; error bars reflect SD.

Our results thus far suggest that the global resistance to apoptosis during *Drosophila* development is likely mediated by transcriptional mechanisms that limit expression of proapoptotic genes during resistant stages. Given that HDACs are critical mediators of transcriptional repression, we tested if HDACi had the ability to alter the programmed resistance to apoptosis observed during larval stages. The HDACi TSA has previously been shown to have low toxicity when fed to *Drosophila* larvae at 10 µM (Pile *et al.* 2001). At these low doses of TSA, there is an approximately twofold increase in the levels of several proapoptotic genes in eL3 animals (Figure 3A). Although eL3 animals are extremely resistant to *reaper* (Figure 3B, blue line), feeding 10 µM TSA results in more than a twofold increase in sensitivity. Of these TSA-fed eL3 animals, ∼39% eclose after *reaper* treatment compared to 88% of eL3 that eclose without TSA feeding. We also tested the effect of TSA feeding in *dronc* null mutant animals. These mutant animals cannot initiate a caspase cascade (Chew *et al.* 2004) and, as expected, *dronc* mutant eL3 animals were highly resistant to *reaper* treatment (Figure 3C, blue line). However, feeding TSA did not significantly enhance the sensitivity to *reaper* in the absence of *dronc* activity (Figure 3C, red line), demonstrating that TSA-mediated enhancement of lethality is caspase-dependent and thus reflects an increased likelihood for triggering apoptosis.

Given the partial relief of resistance with 10 µM TSA, we tested if the disruption of programmed resistance depended on the dose of either HDACi or apoptotic stimulus. Increasing the expression of *reaper* to ∼350-fold (see *Materials and Methods*) starts killing a small fraction of eL3 animals (Figure S2 in File S1, blue line); combining this higher dose of *reaper* with 10 µM TSA results in lethality of nearly all eL3 animals (Figure S2 in File S1, red line). In addition to TSA, SAHA also relieves resistance to apoptosis in eL3 animals, and it does so in a dose-dependent manner (Figure S3 in File S1). Taken together, these results suggest that proapoptotic treatments together with low doses of HDACi are sufficient to reverse developmentally programmed resistance to apoptosis.

### HDAC1 is a critical regulator of global resistance to apoptosis

**Figure 4:**
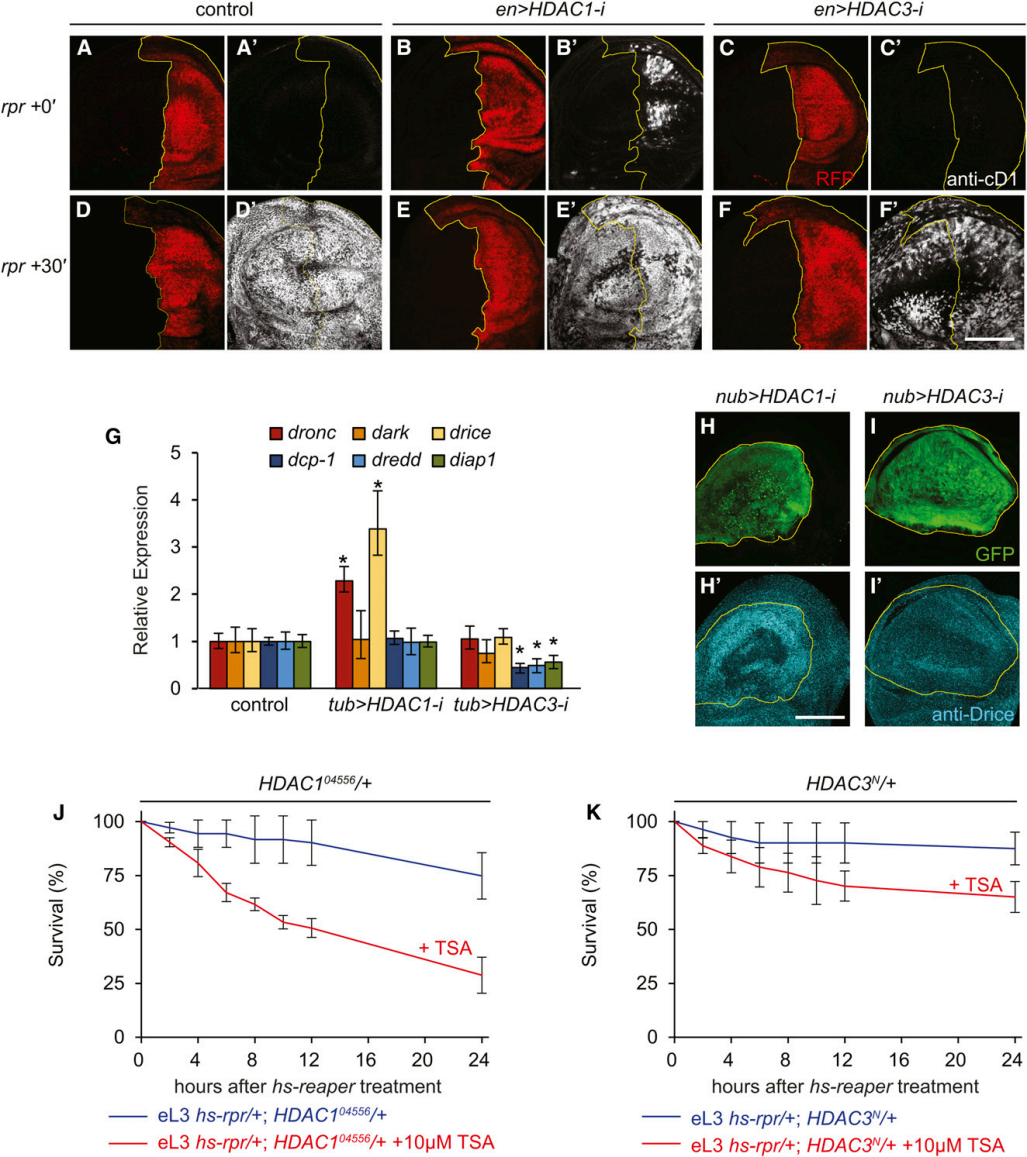
HDAC1 and TSA act synergistically to relieve programmed resistance to apoptosis. (A–F’) Caspase activation immediately (A–C’) or 30 min after (D–F’) *reaper* treatment in wing imaginal discs dissected from wL3 animals with RNAi knockdown of either *HDAC1* or *HDAC3*. Caspase activation detected by staining with antibodies directed to cleaved Dcp-1 (anti-cD1; in white). Region of RNAi expression is defined by the *en-Gal4* driver, shown by RFP expression (in red) and outlined in yellow. All images captured with the same imaging parameters. Bar, 100 µm. (G) Expression levels of proapoptotic genes after whole animal RNAi of either *HDAC1* or *HDAC3*. The whole animal RNAi knockdown was achieved with the *tub-Gal4* driver, and the apoptotic genes examined are the same as those in Figure 1. qPCR results reflect triplicate biological samples, represented relative to the levels of *rp49* and normalized to levels in control eL3 animals. Error bars calculated by Relative Expression Software Tool analysis. *p < 0.05. (H–I’) Change in expression of Drice protein in wing discs with RNAi knockdown of either *HDAC1* or *HDAC3*. Drice protein levels detected by staining with antibodies directed to Drice (anti-Drice, in cyan). Region of RNAi expression is defined by the *nub-Gal4* driver, shown by GFP expression (in green) and outlined in yellow. Bar, 100 μm. (J and K) The effect of TSA feeding on survival in response to *reaper* expression in eL3 animals that are heterozygous for a null mutation in either *HDAC1* (J) or *HDAC3* (K). Genotypes are shown below each of the graphs. Each condition was tested in triplicate with at least 25 animals each; error bars reflect SD.

In order to identify an HDAC that mediates the HDACi-dependent effects on resistance to apoptosis, we examined the role of class I HDACs. Class I HDACs, which include *HDAC1/Rpd3* and *HDAC3* in *Drosophila*, are susceptible to inhibition by both TSA and SAHA (de Ruijter *et al.* 2003; Cho *et al.* 2005). Thus, we used RNA interference (RNAi) to knockdown these genes in wing imaginal discs to determine their role in programmed resistance to apoptosis. Wing discs expressing RNAi in the posterior compartment of the wing disc with the *en-Gal4* driver were dissected immediately or 30 min after *reaper* treatment. Dissected tissues were then stained with antibodies directed to cleaved Dcp-1 (anti-cD1) to detect caspase activation. As expected, and consistent with what is observed for anti-cC3 staining, control tissues do not show activated caspases until 30 min after *reaper* treatment (Figure 4, A and D). In contrast, knockdown of *HDAC1* shows precocious activation of caspases immediately after *reaper* treatment and an enhanced activation 30 min later (Figure 4, B and E); knockdown of *HDAC1* alone, without *reaper* treatment, does not result in caspase activation (Figure S4 in File S1). These results indicate that loss of *HDAC1* does not directly trigger caspase activation—it instead increases the sensitivity to apoptotic triggers. Knockdown of *HDAC3*, on the other hand, does not appear to alter the time course of caspase activation after *reaper* treatment (Figure 4, C and F). Moreover, whole animal knockdown of *HDAC1* (with the *tub-Gal4* driver) results in increased expression of *dronc* and *drice* (Figure 4G), while similar knockdown experiment with *HDAC3* shows a reduction in levels of *dcp-1*, *dredd*, and *diap1*, but no effects on levels of *dronc*, *dark*, or *drice*. Consistently, knockdown of *HDAC1* but not *HDAC3* increased levels of Drice protein (Figure 4, H and I). These results suggest that HDACs play distinct roles in regulating the levels of proapoptotic genes. Importantly, these results show that *HDAC1* is required in a cell-autonomous manner during programmed resistance to apoptosis.

To further examine the role of *HDAC1* in programmed resistance to apoptosis, we examined the ability to survive *reaper* treatment in *HDAC1* and *HDAC3* mutant animals. Animals carrying null mutations in either of these genes die early due to their essential role during embryogenesis (Chen *et al.* 1999; Zhu *et al.* 2008). In order to circumvent this limitation, we tested the resistance to apoptosis in eL3 animals heterozygous for null mutations in either *HDAC1* or *HDAC3*. Animals heterozygous for these mutations appear healthy and viable. However, loss of one copy of *HDAC1* increased the sensitivity to *reaper* treatment, with ∼75% of eL3 animals surviving after 24 hr (Figure 4J; *cf*. Figure 3B). Feeding 10 µM TSA had a synergistic, proapoptotic effect on *HDAC1* heterozygous animals, resulting in a strong reversal of the programmed resistance to apoptosis in these animals (Figure 4J). On the other hand, animals heterozygous for *HDAC3* are indistinguishable from controls in their response to *reaper*, with or without TSA (Figure 4K; *cf*. Figure 3B). Taken together, these results demonstrate that HDACs like HDAC1 play a critical role in the establishment and/or maintenance of the programmed resistance to apoptosis during *Drosophila* development.

## Discussion

In this study, we exploit the developmentally programmed resistance to apoptosis in *Drosophila* to understand the relationship between levels of proapoptotic genes, pharmacological inhibitors of HDAC activity, and the ability to trigger apoptosis. Our results indicate that transcriptional control of proapoptotic genes plays a critical role in the programmed resistance during the *Drosophila* life cycle. Stages that are resistant to apoptosis have about fivefold lower levels of critical proapoptotic genes. Increasing levels of proapoptotic genes sensitizes resistant stages, while decreasing their levels in sensitive stages confers resistance. We also show that HDAC1 plays a critical role in resistance and that HDACi can effectively relieve the developmentally programmed resistance to apoptosis. Finally, our results suggest that this developmentally programmed resistance in *Drosophila* is a good experimental model for studying the mechanisms that allow malignant cells to avoid apoptosis.

Although the relationship between high levels of proapoptotic genes and increased likelihood of triggering apoptosis has been empirically established, most of the available evidence in mammals and simpler organisms (Ni Chonghaile *et al.* 2011; Florentin and Arama 2012) presents a static view of apoptotic thresholds. In this static view, differences in the starting levels of proapoptotic genes predetermine the probability of initiating apoptosis in a specific cell type. However, the starting conditions are not static; instead, it is becoming apparent that they are dynamically regulated; for example, during mammalian neurogenesis, dynamic control of *Apaf-1* levels dictates changes in sensitivity to apoptosis (Wright *et al.* 2004, 2007). We observe similar changes in apoptotic gene expression levels during *Drosophila* development, suggesting an underlying conserved mechanism. Our data suggest that this conserved mechanism involves the coordinate transcriptional repression, through HDAC activity, of critical proapoptotic genes which, in turn, generate dramatic differences in the ability to initiate apoptosis.

Our data show that pharmacological inhibitors of HDAC activity in *Drosophila*, as in humans, promote apoptosis by increasing levels of proapoptotic genes, thereby reducing apoptotic thresholds. Even relatively low, nontoxic levels of HDACi reduce apoptotic thresholds enough to disrupt programmed resistance to apoptosis, sensitizing previously resistant tissues. Consistently, although HDACi are sometimes used in monotherapies in the clinic, they appear to be more effective in drug cocktails with cytotoxic agents that trigger apoptosis (Frew *et al.* 2009). These parallels between clinical outcomes and the effects of similar treatments on programmed resistant tissues during development further reflect the likelihood of shared mechanisms during normal and abnormal physiology. Moreover, these similarities also demonstrate that the therapeutic outcomes of HDACi treatment are not, as current literature suggests, selective to tumor cells. In other words, healthy cells normally protected by programmed resistance may, in the presence of HDACi, become more sensitive to therapeutic and environmental cytotoxic agents. Given that we do not know if the effects of HDACi treatment on the epigenetic mechanisms that mediate programmed resistance are reversible, the potential long-term consequences of HDACi-based therapies on other tissues in the body remain to be explored.

The potential long-term consequences of HDACi treatment are further complicated by the fact that the function of programmed resistance to apoptosis during development remains unknown. Programmed resistance may help protect critical and/or long-lived cells because the value of these cells, even if slightly damaged, outweighs the consequences of their loss. In mammals, mature neurons, which need to survive for the entire life span of the animal, likely require this protection (Kole *et al.* 2013). In addition, very recent results suggest that resistance to apoptosis may play a critical role during tissue regeneration and homeostasis. In planaria, for example, suppression of apoptosis appears to be required to facilitate net growth of regenerating tissues (LoCascio *et al.* 2017). A similar suppression of apoptosis occurs during pathogen-induced proliferation in the *Drosophila* gut (Loudhaief *et al.* 2017). Whether the suppression of apoptosis during tissue homeostasis involves coordinated changes in levels of proapoptotic genes or whether HDACi treatment disrupts these processes is yet to be determined. Importantly, given that we have yet to fully appreciate the role of programmed resistance to apoptosis during development, therapeutic interventions that can disrupt this process in healthy tissues should be used with caution.

## Supplementary Material

Supplemental material is available online at www.g3journal.org/lookup/suppl/doi:10.1534/g3.117.041541/-/DC1.
