# Supplementary material for: HDAC Inhibitors Disrupt Programmed Resistance to Apoptosis During Drosophila Development
Source: G3 (Bethesda). 2017 Apr 27;7(6):1985–93. doi: 10.1534/g3.117.041541 (PMC5473774; doi:10.1534/g3.117.041541)
Supplement: Supplementary file 1 [file 1985File001.pdf]

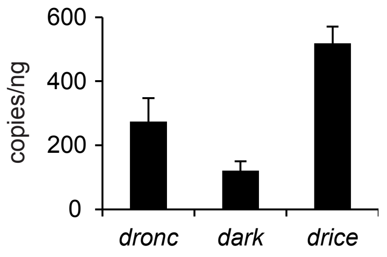

Figure S1. Absolute quantification of *dronc*, *dark*, and *drice* in eL3 animals. *y*-axis represents number of transcripts of target gene per ng of total RNA. qPCR results reflect triplicate biological samples and error bars represent standard deviation.

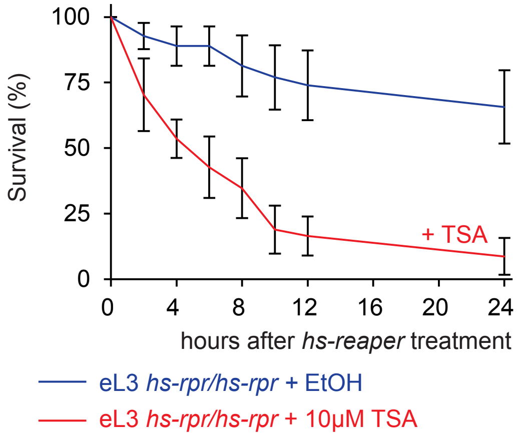

Figure S2. A low dose of HDACi with a high dose of *reaper* overcomes the programmed resistance in eL3 animals. eL3 animals carrying two copies of the *hs-rpr* transgene generate a ~350-fold induction of *reaper* (Kang and Bashirullah 2014). At these high doses or *reaper* expression, some control eL3 animals start dying; feeding 10μM TSA to these animals is sufficient to overcome the resistance to apoptosis. Each condition tested in triplicate with at least 25 animals each; error bars reflect standard deviation.

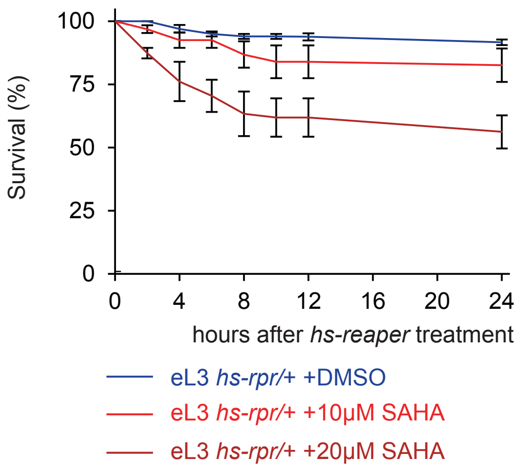

Figure S3. SAHA relieves programmed resistance in eL3 animals in a dose-dependent manner. *reaper*-treated eL3 animals fed 10μM SAHA have a slightly increased rate of lethality compared to control animals fed DMSO. This lethal effect of *reaper* is enhanced in eL3 animals fed 20μM SAHA. Each condition tested in triplicate with at least 25 animals; error bars reflect standard deviation.

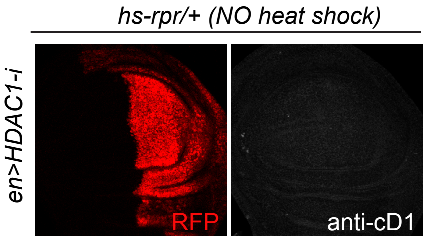

Figure S4. Knockdown of *HDAC1* is not sufficient to trigger caspase activation. In the absence of *reaper*-treatment, knockdown of *HDAC1* in wL3 wing imaginal discs (*en>HDAC1-i*) does not show any caspase activation. Region of RNAi expression is defined by the *en-Gal4* driver, shown by RFP expression (in red, left panel). Caspase activation detected by staining with antibodies directed to cleaved Dcp-1 (anti-cD1; in white, right panel).

Table S1. Stage-specificity of survival rates after *reaper* treatment during third instar development.

Stage	24 h Survival (%)	Eclosion Rate (%)
72-76 h AEL	98.33 ± 2.89	95 ± 8.6
88-92 h AEL	90.97 ± 6.64	81.12 ± 12.65
0-4 h ASE	86.27 ± 13.40	63.29 ± 10.18
4-8 h ASE	92.29 ± 3.66	60.26 ± 9.07
8-12 h ASE	64.49 ± 22.38	27.76 ± 10.24
12-16 h ASE	25.60 ± 21.15	11.81 ± 2.57
16-20 h ASE	0 ± 0	0 ± 0
20-24 h ASE	0 ± 0	0 ± 0

Each stage was tested in triplicates with at least 25 animals each; ranges reflect standard deviation. 24 h survival rates are plotted in Figure 1B. AEL, after egg-lay; ASE, after Sgs3-GFP expression.

Table S2. Effect of loss of copy number in *dronc*, *drice*, and/or *dark* on survival after *reaper* treatment during wL3 development.

Alleles (with *hs-rpr/+*)	24 h Survival (%)	Eclosion Rate (%)
+/+	0 ± 0	0 ± 0
dronc^{51}*/Df*	98.7 ± 2.3	*n.a.*
drice$^{\Delta 1}$*/Df*	97.3 ± 2.3	*n.a.*
dronc^{51}*/+*	15.7 ± 6.2	0 ± 0
dark^{82}*/+*	11.1 ± 4.8	0 ± 0
drice$^{\Delta 1}$*/+*	16.8 ± 16.9	0 ± 0
dark^{82}*/+;dronc*^{51}*/+*	69.4 ± 2.3	38.8 ± 1.8
dronc^{51}*/drice*$^{\Delta 1}$	73.4 ± 3.7	31.6 ± 11.1
dark^{82}*/+;drice*$^{\Delta 1}$*/+*	78.7 ± 0.6	29.8 ± 0.9
dark^{82}*/+;dronc*^{51}*/drice*$^{\Delta 1}$	87.5 ± 3.5	64.0 ± 1.7

Each condition tested in triplicates with at least 25 animals each; ranges reflect standard deviation. 24 h survival rates included in Figure 2A. *n.a.*, *dronc* and *drice* mutant animals are lethal and do not eclose.

Table S3. Sequences and source of primers used for qPCR analysis.

Gene	Primer sequence	Source
rp49 F	CCAGTCGGATCGATATGCTAA	(Denton *et al.* 2009)
rp49 R	ACGTTGTGCACCAGGAACTT	
dark F	TGCACTTCATCGGAGTATCG	(Denton *et al.* 2009)
dark R	AGATCGATCCACTGGCATTC	
dronc F	CTCGCTAAACGAACGGAGAAC	(Ihry *et al.* 2012)
dronc R	CAACGACACCCACATAAGGG	
drice F	CAGGGCGACAGATTGGATGG	(Kang and Bashirullah 2014)
drice R	GCCGCGGGTGGTGTTGC	
dcp-1 F	CTGGAGAAGGGCGTTACCGAGAC	(Kang and Bashirullah 2014)
dcp-1 R	ATTGTTGATGTTGCGCCAGGAGA	
dredd F	ATACAAGCCTGCCAGGAGAA	(Denton *et al.* 2009)
dredd R	AGGATATCGGCGATGTGTTC	
diap1 F	CACACAGGCTTCAGAGGAAGAG	(Kang and Bashirullah 2014)
diap1 R	CTGCTGTTTGTCGAGGGAGTAG	
